# *Alpha2beta1* Integrin Polymorphism in Diffuse Astrocytoma Patients

**DOI:** 10.3389/fonc.2022.914156

**Published:** 2022-07-22

**Authors:** Silvia A. Teixeira, Regislaine V. Burim, Mariano S. Viapiano, Lucas T. Bidinotto, Suely K. Nagashi Marie, Suzana M. Fleury Malheiros, Sueli M. Oba-Shinjo, Augusto F. Andrade, Carlos G. Carlotti

**Affiliations:** ^1^ Department of Surgery and Anatomy, Ribeirão Preto Medical School, University of São Paulo (USP), São Paulo, Brazil; ^2^ Molecular Oncology Research Center, Barretos Cancer Hospital, Barretos, São Paulo, Brazil; ^3^ Department of Clinical, Toxicological and Bromatological Analysis, University of São Paulo (USP), Faculty of Pharmaceutical Sciences of Ribeirão Preto, São Paulo, Brazil; ^4^ Department of Neurosurgery, Brigham and Women’s Hospital and Harvard Medical School, Boston, MA, United States; ^5^ Department of Neuroscience and Physiology, SUNY Upstate Medical University, Syracuse, NY, United States; ^6^ Department of Pathology, School of Medicine, UNESP– Univ. Estadual Paulista, Botucatu, Brazil; ^7^ Barretos School of Health Sciences, Dr. Paulo Prata – FACISB, Barretos, Brazil; ^8^ Department of Neurology, Medical School, University of São Paulo (USP), São Paulo, Brazil; ^9^ Department of Neurology, Faculty of Medicine, Federal University of São Paulo (UNIFESP), São Paulo, Brazil; ^10^ Department of Internal Medicine, Faculty of Medicine, University of São Paulo (USP), São Paulo, Brazil; ^11^ Department of Human Genetics, McGill University, Montreal, QC, Canada

**Keywords:** single nucleotide polymorphism, extracellular matrix, brain microenvironment, tumor progression, low grade glioma, invasion, ITGA2

## Abstract

Integrins are heterodimeric transmembrane glycoproteins resulting from the non-covalent association of an α and β chain. The major integrin receptor for collagen/laminin, α2β1 is expressed on a wide variety of cell types and plays an essential role in the adhesion of normal and tumor cells to the extracellular matrix. Integrin-triggered signaling pathways promote the invasion and survival of glioma cells by modifying the brain microenvironment. In this study, we investigated the association of a specific genetic polymorphism of integrin *α2β1* with the incidence of diffusely infiltrating astrocytoma and the progression of these tumors. Single-nucleotide polymorphism in intron 7 of the integrin *ITGA2* gene was examined in 158 patients and 162 controls using polymerase chain reaction and restriction enzyme analysis. The *ITGA2* genotype +/+ (with a *Bgl*II restriction site in both alleles) exhibited higher frequency in grade II astrocytoma compared to control (P = 0.02) whereas the genotype -/- (lacking the *Bgl*II site) correlated with the poorest survival rate (P = 0.04). In addition, *in silico* analyses of *ITGA2* expression from low-grade gliomas (LGG, n = 515) and glioblastomas (GBM, n = 159) indicated that the higher expression of *ITGA2* in LGG was associated with poor overall survival (P < 0.0001). However, the distribution of integrin *ITGA2 Bgl*II genotypes (+/+, +/-, -/-) was not significantly different between astrocytoma subgroups III and IV (P = 0.65, 0.24 and 0.33; 0.29, 0.48, 0.25, respectively) compared to control. These results suggest a narrow association between the presence of this SNP and indicate that further studies with larger samples are warranted to analyze the relation between tumor grade and overall survival, highlighting the importance of determining these polymorphisms for prognosis of astrocytomas.

## Introduction

Gliomas constitute the most common primary brain tumors and include several histologically subtypes, most of them malignant and highly invasive (1). High-grade gliomas including glioblastoma (GB) are considered among the most devastating cancers due to their markedly short post-diagnosis survival time (2), accounting for approximately 15% of all intracranial neoplasms and 60%–75% of astrocytic tumors (3, 4).

A hallmark of the severity of astrocytomas is the ability of tumor cells to invade and infiltrate the brain parenchyma (5). Although the molecular mechanisms underlying these events remain to be better elucidated, the first step encompasses the adhesion of tumor cells to the host’s extracellular matrix (ECM), which is mediated primarily by integrins on the tumor cell surface (6). Aggregation of integrin receptors followed by association of cytoskeletal proteins and tyrosine kinase-mediated phosphorylation are key events responsible for diverse cell responses such as cell migration and differentiation, tissue remodeling, cell proliferation, angiogenesis, and tumor cell invasion, metastasis, and survival (7–11).

Several integrin subunits are significantly upregulated in GB compared to normal brain tissue (12) including α2, α3, α4, α5, α6, and β1 integrins (11, 13–15). Integrin expression and ECM production are directly correlated with the tumor grade and have been found to promote human glioma progression and reduce survival (15–17).

Integrins are transmembrane glycoproteins with non-covalently associated and chains, which mediate the interaction of tumor cells with their microenvironment (17–19). The association of distinctive and subunits determines the functional specificity of receptors (11). Eight different β chains and 18 α chains have been described, whereas only 24 different heterodimers have been observed (7, 19, 20). Among these, α2β1 integrin is a major collagen receptor that plays an essential role in the adhesion of normal and tumor cells to the extracellular matrix (20–23).

The gene encoding integrin *α2* chain (*ITGA2*) is described in the occurrence and progression of multiple cancers, including colorectal cancer, lung cancer, and breast cancer (24, 25). ITGA2 mediated adhesion to type I collagen and are expressed on cancer cells, immune cells, stroma cells, and endothelial cells (9, 21, 26). The high expression in tumor tissue identified ITGA2 as a potential therapeutic target to treatment of cancer. In addition, ITGA2 have been used as a novel molecular target to treat GBM; nevertheless, its role in diffuse glioma still remains to be elucidated (27). However, changes in ITGA2 expression may affect therapeutic targets, immune microenvironment, and the immunogenicity of glioma tumors (27, 28).

*ITGA2* has several single-nucleotide polymorphisms (SNPs) including the *Bgl*II polymorphism (29, 30). A/G SNP in intron 7 creates a restriction site for the enzyme *Bgl*II (sequence AGATCT, *Bgl*II +/-) (31). This polymorphism is in linkage disequilibrium, and the *Bgl*II (+) allele is linked to the 807T/873A allele. *Bgl*II (-) is linked to the 807C/873G allele. The *Bgl*II(+) allele has been associated with a high level of *ITGA2*, and *Bgl*II (-) was associated with a low level (29–31). Additionally, the *ITGA2 Bgl*II polymorphism has been associated with increased risk for higher disease stages of breast cancer (32), positive history for oral cancer (33), and prevalence of diabetic retinopathy, myocardial infarction, and stroke (34–36).

Here, we hypothesize that the functional *ITGA2 Bgl*II polymorphism of integrin α2β1 can also correlate with the incidence or progression of glioma tumors. To test this hypothesis, the prevalence of this polymorphism was evaluated in patients with diffuse infiltrating astrocytomas (malignant grades II to IV) compared to a healthy control group. Our results indicate that *Bgl*II polymorphisms of *ITGA2* have increased frequency in grade II astrocytoma cases and suggests a protective effect on the risk to LGG; however, more studies are necessary to correlate the polymorphism with tumor progression and overall survival.

## Materials and Methods

### Subject ´ Recruitment

The study involved 320 unrelated individuals with similar ethnic backgrounds, 158 patients with astrocytomas, and 162 age- and sex-matched controls without histories of cancer or other major disease. Individuals were recruited at the Clinical Hospital of Ribeirão Preto Medical School, University of São Paulo (HC-FMRPUSP), Clinical Hospital of the Medical School, University of São Paulo (HC-FMUSP), and São Paulo Hospital, Federal University of São Paulo (UNIFESP) during the Cancer Clinical Genome Project (FAPESP 01/13716-7, 03/00960-2, 04/12133-6).

Tumor cases and controls were distributed as 97 men/61 women and 97 men/65 women, respectively. The distribution of the 158 patients with astrocytoma was as follows: 28 with grade II, 26 with grade III, and 104 grade with IV astrocytoma (WHO 2016). The control group consisted of individuals who lived in the study areas for at least 1 year with any known history of cancer or chronic disease in a self-reported questionnaire. On the basis of phenotype characteristics and family history, 120 patients and 113 controls were identified as white (European descendants); 27 patients and 27 controls as mulatto, and 8 patients and 13 control as black (African descendants); 2 and 8 were oriental descendants; and 1 and 1 were classified as others. Epidemiological data from the study population were obtained by a standard interviewer-administered questionnaire, including data on social habits, health problems, and ancestry. The human subject protocol was approved by the local Institutional Review Boards of the participating institutions; written informed consent was obtained from all subjects or their parents.

### Genotyping

**Figure 1:**
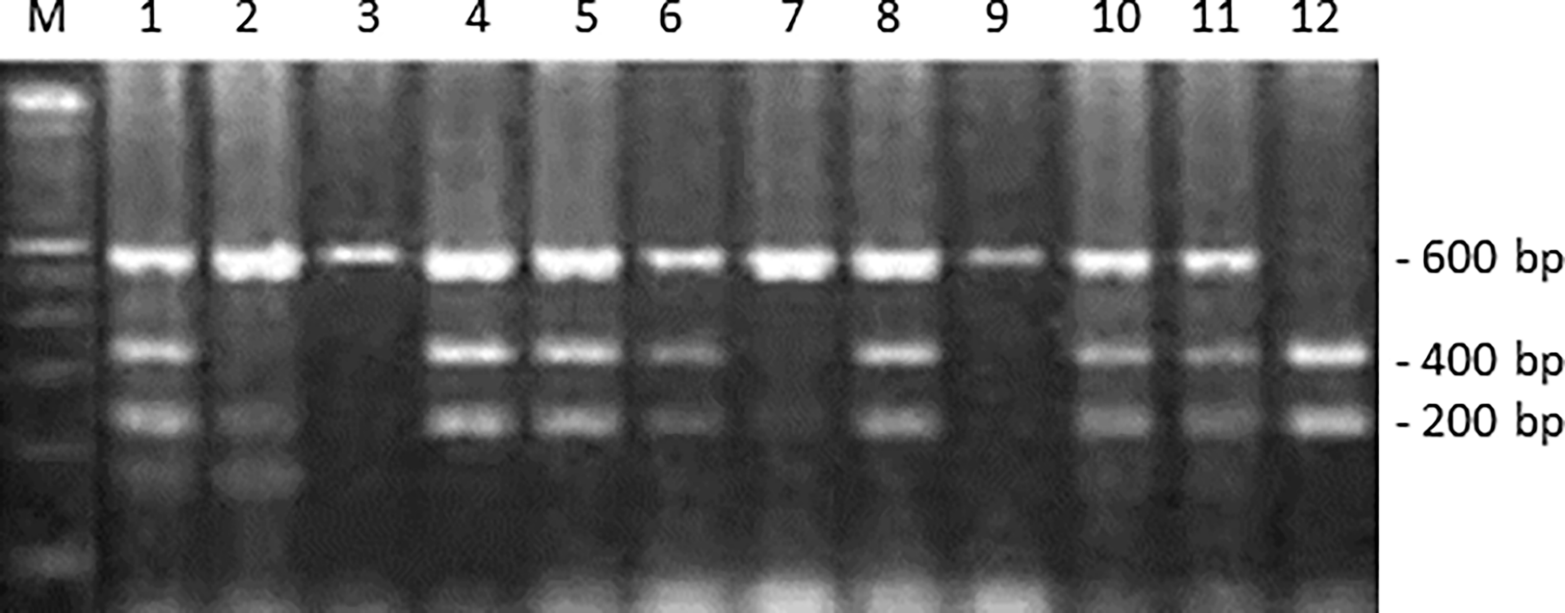
*ITGA2* polymorphism genotyping by PCR-RFLP assay. This panel shows the *Bgl*II digestion of *ITGA2* gene PCR products. Lane M: 100-bp molecular marker. Lanes 2, 3, 7, and 9 are representative of a homozygote -/- (absence of *Bgl*II site); lanes 1, 4, 5, 6, 8, 10, and 11 of a heterozygote -/+; and lane 12 of a homozygote polymorphic +/+ (presence of *Bgl*II site).

Genomic DNA was extracted from peripheral blood lymphocytes by the conventional phenol-chloroform method. Isolated DNA was resuspended in Tris–EDTA buffer (pH 8.0) and stored at -20°C until use. Polymerase chain reaction-restriction fragment length polymorphism (PCR-RFLP) to determine the *ITGA2* genotype using *Bgl*II (New England Biolabs, Beverly, MA) was carried out as previously described [26]. The PCR primers used were as follows (5′–3′): forward, GATTTAACTTTCCCGACTGCCTTC (nucleotide number 2789-2812); reverse, CATAGGTTTTTGGGGAACAGGTGG (nucleotide number 3346-3369, GenBank accession number AF035968). Restriction fragments were separated by electrophoresis using 2% agarose gels. The PCR products containing a *Bgl*II (+) site yielded fragments of 200 and 400 bp upon enzymatic digestion, as shown in **Figure 1**.

### *In Silico* Analysis of *ITGA2* Expression in TCGA Dataset

There were obtained normalized RNASeq and clinicopathological data from The Cancer Genome Atlas (TCGA) from low-grade gliomas (LGG, n = 515) and glioblastomas (GBM, n = 1) using the RTCGA Toolbox package (37) on R. The number of reads of *ITGA2* was log10-transformed. Then, patients were distributed into two groups according to the median. Those with the number of reads equal or higher to the median were considered with a high expression. Otherwise, the patients were considered with a low expression.

In LGG, the expression of ITGA2 was associated with histology (astrocytoma, oligoastrocytoma, or oligodendroglioma), grade (2 or 3), IDH, TERT, ATRX mutation status, MGMT promoter methylation status, and subtype. Moreover, in GBM, the expression of *ITGA2* was associated with IDH, TERT, ATRX mutation status, MGMT promoter methylation status, and subtype. The expression of ITGA2 was analyzed according to Noushmehr et al. and Cecarelli et al. (38, 39). Additionally, overall survival was analyzed through Kaplan–Meier curves, using the log-rank test to compare the groups based on ITGA2 expression. Finally, hazard ratio and 95% confidence interval were determined using Cox proportional hazards in LGG and GBM using *ITGA2* expression as contrast groups and death as endpoint.

### Statistical Analysis

Differences between groups were compared by chi-square test or Fisher´s exact test. The 95% confidence intervals (95% CI) of the percentage were calculated by assuming a binomial distribution. Sex-age-adjusted odds ratio (ORs) and 95% confidence interval (95% CI) were calculated according to an unconditional logistic model. Goodness of fit of genotype distribution was analyzed by the Hardy–Weinberg equilibrium. Values of P < 0.05 were considered statistically significant. All statistical analyses were performed using the statistical package SPSS.

## Results

The genotype and allele frequency for *ITGA2 Bgl*II polymorphism in the tumor and control groups are shown in **Table 1**. The frequencies for the three possible genotype frequencies (+/+, +/-, and -/-) were in agreement with those predicted by the Hardy–Weinberg equilibrium. Genotype and allelic frequency were compared between tumor cases and controls as well as within the subgroups of astrocytomas (WHO grade II, III, and IV) (**Table 2** and **Supplementary Tables S1, S2**).

**Table 1:** Distribution of allele and genotype frequencies of polymorphisms and statistical data obtained from the analysis of integrin *α2 BglII* polymorphisms in astrocytoma grades II, III, and IV and control.

	Controls	Grade II astrocytoma	Grade III astrocytoma	Grade IV astrocytoma	Astrocytomas total
	N=162	N=28	N=26	N=104	N=158
***Integrin α2 BglII***					
***–/–***	76 (46.9)	8 (28.6)	10 (38.5)	41 (39.4)	59 (37.5)
***–/+***	73 (45.1)	14 (50.0)	12 (46.2)	53 (50.9)	79 (50)
***+/+***	13 (8.0)	6(21.4)	4 (15.4)	10 (9.6)	20 (12.7)
***+/–*** *+* ***+/+***	86 (53.1)	20(71.4)	16 (61.5)	63 (60.6)	99 (62.7)
**Allele frequency**	0.69	0.54	0.62	0.65	0.62
***–***	0.31	0.46	0.38	0.35	0.38
***+***					

-/-, homozygous for the wild-type allele; -/+ heterozygous; +/+, homozygous for the polymorphic allele.

**Table 2:** Distribution of allele and genotype frequencies of polymorphisms and statistical data obtained from the analysis of integrin *α2 BglII* polymorphisms in the astrocytoma grade II and control.

Number (%)				
	Grade II astrocytoma	Controls	OR (95% CI)	P
***Integrin* α2β1 *Bgl*II**				
*–/–*	8/28 (28.6)	76/162 (46.9)	1.0 (ref.)	0.25
*+/–*	14/28 (50.0)	73/162 (45.1)	1.82 (0.72–4.60)	**0.02^a^**
*+/+*	6/28 (21.4)	13/162 (8.0)	4.38 (1.31–14.72)	0.09
*+/–* + +*/+*	20/28 (71.4)	86/162 (53.1)	2.21 (0.91–5.31)	0.47
**Alleles**	0.54	0.69	–	
*–*	0.46	0.31	–	
+				

-/-, homozygous for the wild-type allele; -/+, heterozygous; +/+ homozygous for the polymorphism allele.

^a^ The homozygous integrin α2β1 BglII +/+ variant was significantly more prevalent in grade II astrocytoma patients than in control subjects (P = 0.02). Bold values are statistically significant.

The frequencies of *ITGA2 Bgl*II polymorphism observed in control subjects were in agreement with those reported for other populations (**Table 1**) (36, 40). Multivariate analysis did not reveal an association of this polymorphism with age, race, or gender as co-variable (**Supplementary Tables S3, S4**). In contrast, male patients with astrocytoma showed higher frequencies than women for the *ITGA2 Bgl*II +/+ and +/- genotypes; however, these differences were not statistically significant.

**Figure 2:**
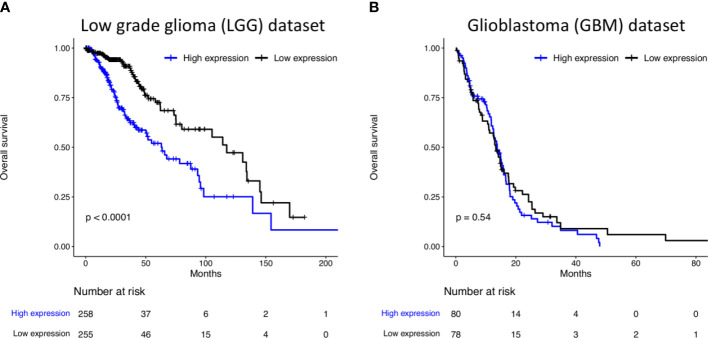
Kaplan–Meier curves of survival related to *ITGA2* in **(A)** 515 patients LGG. LGG patients with high expression of *ITGA2* had a poorer overall survival compared with patients with low expression and **(B)** 159 patients with GBM. The expression of *ITGA2* in GBM was not statistically significant when compared with patients with low and high expressions and overall survival.

Based on TCGA database, we analyzed the expression of *ITGA2* on gliomas. The analyses demonstrated that, in LGG, oligodendrogliomas were associated with low *ITGA2* expression as well as lower-grade tumors (Grade 2). Additionally, low *ITGA2* expression was associated with mutation in IDH, methylation in MGMT and subtype IDHmut-codel or codel, and mesenchymal-like tumors (**Supplementary Table S5**). Similarly, in GBM, low *ITGA2* expression was associated with mutation in IDH. Moreover, low *ITGA2* expression was associated with absence of mutation in TERT and G-CIMP and proneural tumors (**Supplementary Table S6**). Finally, a poor overall survival was found in LGG patients with high expression of *ITGA2* (**Figure 2**, P < 0.0001 in log rank test), presenting a hazard ratio of 2.361 (95CI 1.633–3.412, P < 0.001 in Cox proportional hazards test, **Supplementary Table S7**). No difference was found regarding the overall survival of GBM patients considering *ITGA2* expression (**Figure 2** and **Supplementary Table S7**).

**Figure 3:**
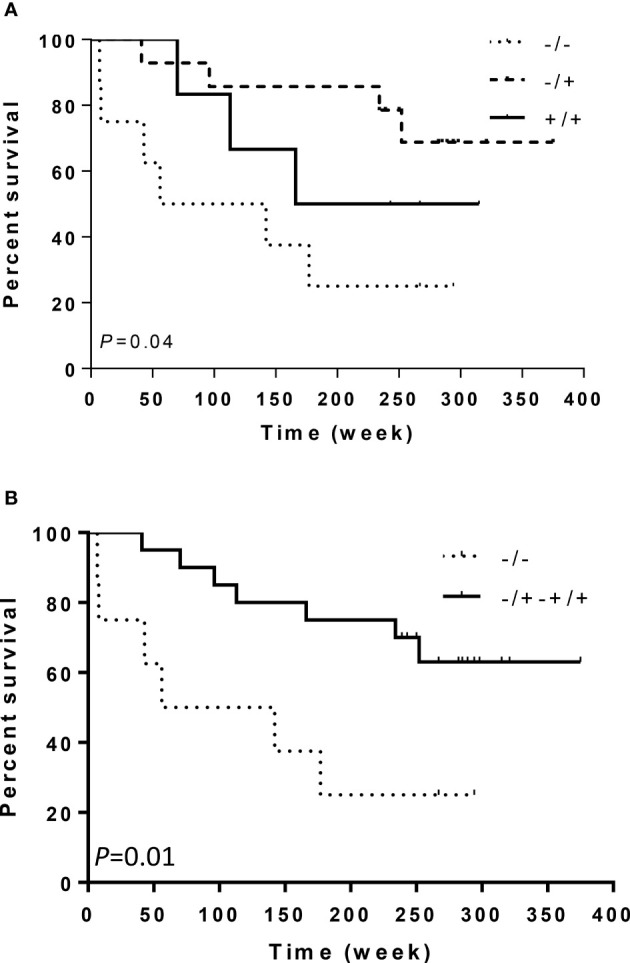
Kaplan–Meier curves of survival related to *ITGA2* polymorphisms in 28 patients with astrocytoma grade II. Patients with *ITGA2* -/- had a poorer prognosis than patients with **(A)** *ITGA2* +/+ or **(B)** *ITGA2* +/+,+/-.

Our results revealed that the *ITGA2 Bgl*II +/+ genotype was significantly overexpressed in patients with grade II astrocytoma compared to controls, OR (95% CI) 4.38 (1.31–14.72) and P = 0.02 (**Table 2**). However, the overrepresentation of this genotype did not extend to the astrocytoma subgroups III and IV (P = 0.65, 0.24, and 0.53; 0.29, 0.48, 0.25, respectively), suggesting a narrow association between the presence of this SNP and tumor grade (**Table 1** and **Supplementary Table S1**). Also, there were no significant differences in the frequencies of these genotypes when comparing each tumor grade against the other grades (**Supplementary Table S2**). Although the *ITGA2 Bgl*II +/+ polymorphism was overrepresented in grade II astrocytomas, it was associated with increased survival profile in this group. Survival curves for the three *ITGA2* genotypes were significantly different for *ITGA2 Bgl*II +/+ and +/+,+/- genotypes (P = 0.04 and P = 0.01, respectively) (**Figures 3A, B**). However, the *ITGA2 Bgl*II polymorphism did not show a statistically significant correlation with survival of higher-grade astrocytomas (P > 0.05).

## Discussion

Adhesive molecules of the integrin family, including integrin α2β1, integrin α2β3, and GP Ibα (specially integrins), are critical for adhesion of tumor cells and participate in the cellular mechanisms of tumoral progression (41–44). The subunit of integrin ITGA2 was found to be upregulated in several types of cancer and has been associated with tumor cell proliferation, migration, invasion, and angiogenesis (45–47). In breast cancer, ITGA2 has been linked to tumor aggressiveness, cancer progression (*via* EMT—epithelial–mesenchymal transition), and metastatic ability (47). Interestingly, Guo et al. described that in human GBM cells, *ITGA2* expression is higher than epidermal growth factor receptor (EGFR), an important target in GBM. In addition, they described that ITGA2 was less expressed on normal glial cells, then they suggested ITGA2 as a novel GBM-specific target (27). Nonetheless, the role of ITGA2 in glioma tumor is still not well understood. Furthermore, the high expression in several carcinoma cells of epithelial origin indicates that ITGA2 is an important key pathway in cancer pathogenesis (48–50). The abnormal expression of ITGA2 has been correlated with unfavorable prognoses in multiple types of cancer. The nucleotide polymorphisms affecting adhesive molecules have been associated with the risk and prognosis of astrocytoma tumors (51).

In this study, we have found that a specific polymorphism in the gene coding for a chain of integrin α2β1 (corresponding to the *ITGA2 Bgl*II site) was associated with differences in survival of patients with low-grade astrocytoma, with one specific variant (*ITGA2 Bgl*II +/+) being overrepresented in grade II astrocytoma cases compared to control individuals. To our knowledge, this is the first study investigating the possible involvement of the integrin *α2β1* genomic variant in the prevalence and overall survival of gliomas. Integrin α2 has been investigated in platelets, where it mediates the adhesion of these cells to collagen and regulates platelet function (11, 52, 53). To other solid tumors, alpha chain integrins are sufficient to mediate adhesion to the luminal side of blood vessels and promote extravasation and angiogenesis (54, 55). In pancreatic cancer, the integrin β1 subunit mediates the tumor cell interaction with the tumor microenvironment and ECM proteins, such as types I and IV collagen, laminin, and fibronectin (56); in breast cancer, colorectal cancer, and lung and hepatocellular carcinoma, integrin has been linked to tumor evolution and aggressiveness (24, 25, 28, 57). The aggressiveness with enhancement of metastasis and stemness of colorectal cancer cells have been associated with the interaction of α2β1 that activates the PI3K/Akt/Snail signaling pathway (19). In addition, the expression levels of integrin α2β1, α2β3, and glycoprotein Ibα are associated with invasiveness of tumor cells (58, 59) and could be relevant to explain the correlation between integrin α2β1 polymorphism and glioma grade (60).

Histological analysis of high-grade glioma indicates that integrins α2β1, α5β1, and α6β1 are strongly expressed compared to normal brain tissue (16, 61). All these integrins share the β1 subunit, which is involved in the adhesion, motility, and invasion of glioma cells *in vitro* (62) as well as tumor dispersion in the brain (63). These data suggest that β1 subunit expression might be related to the clinically invasive phenotype of gliomas. Therefore, the polymorphic genetic variation affecting the expression or function of these adhesive receptors could modify the risk of development and the biological aggressiveness of astrocytomas.

We found that the genotypes of *ITGA2 Bgl*II for gliomas and control individuals were distributed in accordance with the Hardy–Weinberg equilibrium and in agreement with those previously published to Caucasian subjects. In control subjects, the frequencies of *ITGA2 Bgl*II polymorphisms for genotypes -/-, -/+, and +/+ were, respectively, 46.9, 45.1, and 8.0 matching prior reports (36) in diabetic retinopathy and in diabetic patients (40).

The total distribution of *ITGA2 Bgl*II genotypes was not significantly different between astrocytomas and controls. Similar results have been described to other tumors and population and failed to find or increased the risk for tumor progression with *Bgl*II (+) polymorphism (64–67). However, future investigation in larger cohorts of patients, and among various racial groups, will be necessary to further define the role of the *Bgl*II genotype in the pathogenesis of GBM. Regarding grade II astrocytoma, the *ITGA2* genotype (+/+ and +/-) exhibited higher frequency and, in contrast, increased median survival compared to the homozygous -/- genotype. These results suggest that the *Bgl*II polymorphism in the gene which codes for the *α2* chain of integrin *α2β1* may have a protective effect on the risk to low-grade astrocytomas. Furthermore, we analyzed the potential impact of *ITGA2* on the overall survival of glioma grade II, III, and IV patients using the same database (TCGA). The correlation between overall survival and *ITGA2* expression was not statistically significant to grade III and IV of astrocytomas. In contrast, in a recent study the ITGA2 expression was significantly associated with decreased GBM patient survival (27). The difference in the results may be due to the number of patients analyzed in the two studies and highlight the relevance of future investigation in a large cohort of patients.

The poor prognosis for patients with LGG shows the diversity of this malignant glioma; therefore, new treatment strategies are needed to continuously improve the prognosis of LGG patients. Similar results to LGG have been described in a series of systematic analysis (68). In another study, the high expression of *ITGA2* was associated with reduced survival rate of solid cancer (28). The high expression of *ITGA2* has been associated with worse prognosis (68), and studies have shown that multiple epigenetic mechanisms regulate *ITGA2* expression in solid tumors including altered promoter methylation (50, 69, 70). In our results, low *ITGA2* expression indicates an association with mutation in IDH, methylation in MGMT in mesenchymal-like tumors, and absence of mutation in TERT and G-CIMP and proneural tumors. Tumor *ITGA2* expressions have been correlated with hypermethylation in prostate cancer and hypomethylation in breast cancer (71). In the breast cancer subtype, the high expression of ITGA2 was variable and was associated with metastases and poor survival (71). In addition, the high expression of *ITGA2* in tumor tissue indicates *ITGA2* as an important clinical biomarker of poor prognosis in patients (19).

ITGA2 has been targeted by small molecules and antibodies as potential cancer therapies and is in clinical trials (27, 45). Recently, it has been reported that the ITGA-2 antibody inhibits cell migration, impede actin organization, and mediate cell apoptosis (27, 45). The anti-integrin therapeutics for GBM has been described as antiangiogenic, anti-invasion, and antitumor strategies (72). In immune therapy, the inhibition of ITGA2 increased the ratio of tumor-killing lymphocytes and decreased the proportion of immunosuppression-related cells in tumors (28). In another study, the authors reported that ITGA2 increased the PD-L1 expression in multiple types of cancer cells and improved the antitumor efficacy of immune therapy (27). Furthermore, immune checkpoint treatment has become a new method to treat cancer and ITGA2 can bring new insights into targeted immunotherapy and, for patient with LGG, may represent a potential molecular marker for targeted therapy (68). Therefore, it is very pivotal to find a new prognostic biomarker to enhance the treatment of glioma and increase the understanding of glioma treatment.

Our data revealed that the *Bgl*II polymorphism was significantly associated with better overall survival of LGG patient. The overrepresentation of the *ITGA2* +/+ polymorphism in grade II, but not in higher-grade astrocytomas, could be explained by the hypothesis that this polymorphism could have a lower tendency to evolve to higher grades or due to the lower percentage of secondary glioblastoma cases (5%) (1), hampering the identification of genetic abnormalities. Hence, a longer and larger follow-up study would be necessary. Additionally, it remains unclear whether this polymorphism affects the synthesis or function of the integrin α2 chain and how these changes would be involved in the development or progression of low-grade astrocytomas. This correlation will be tested in future gene functional experiments.

In conclusion, our findings suggest that the *Bgl*II polymorphism in the gene which codes for the α2 chain of integrin *α2β1* may have a role in the pathogenesis of low-grade astrocytomas and could help the molecular prognosis of these tumors. In addition, the increased frequency of the *ITGA2 Bgl*II polymorphism in grade II astrocytoma suggests a protective effect on the risk factor to LGG; however, more studies are necessary to correlate the polymorphism with tumor progression and overall survival. We expect that these results will provide a foundation for future research into the association between integrin α2β1 and low-grade glioma and astrocytoma progression in other populations.

## Data Availability Statement

The original contributions presented in the study are included in the article/**Supplementary Material**. Further inquiries can be directed to the corresponding author.

## Ethics Statement

The studies involving human participants were reviewed and approved by Institutional Ethics Committee of Clinical Hospital of Ribeirão Preto Medical School, University of São Paulo. The patients/participants provided their written informed consent to participate in this study.

## Author Contributions

ST and RB designed the study and participated in the sample acquisition. SKNM, SMFM, SO-S, and CC contributed with patient samples and clinical information. ST and RB performed the analysis and interpretation of data and wrote the initial draft of the manuscript. LB performed the ***in silico*** analysis. MV and AA did the critical revisions and wrote sections of the manuscript. All the authors reviewed and approved the final manuscript.

## Funding

This research was funded by FAPESP (Process n04/12133-6; 03/00960-2), FAEPA, CAPES, and LICR grants. Barretos Cancer Hospital, Pio XII Foundation.

## Conflict of Interest

The authors declare that the research was conducted in the absence of any commercial or financial relationships that could be construed as a potential conflict of interest.

## Publisher’s Note

All claims expressed in this article are solely those of the authors and do not necessarily represent those of their affiliated organizations, or those of the publisher, the editors and the reviewers. Any product that may be evaluated in this article, or claim that may be made by its manufacturer, is not guaranteed or endorsed by the publisher.
