# Supplementary material for: Alpha2beta1 Integrin Polymorphism in Diffuse Astrocytoma Patients
Source: Front Oncol. 2022 Jul 22;12:914156. doi: 10.3389/fonc.2022.914156 (PMC9353741; doi:10.3389/fonc.2022.914156)
Supplement: Supplementary file 1 [file DataSheet_1.pdf]

Supplementary Material

Table S1. Distribution of allele and genotype frequencies of polymorphism and statistical data obtained from the analysis of integrin $\alpha 2$ *BglII* for controls and astrocytomas patients (grade II, III or IV):

		Number (%)			
		**Astrocytomas**	**Controls**	**OR (95% CI)**	**P**
	Integrin $\alpha 2\beta 1$ BglII				
	-/-	59/158 (37,3)	76/162 (46,9)	1,0 (ref.)	
	+/-	79/158 (50)	73/162 (45,1)	0,72 (0,45-1,14)	0,20
	+/+	20/158 (12,7)	13/162 (8,0)	0,50 (0,23-1,09)	0,12
All Astrocytomas	*+/- + +/+*	99/158 (62,7)	86/162 (53,1)	0,67 (0,43-1,05)	0,11
	Alleles				
	-	0,62	0,69	-	
	+	0,38	0,31	-	0,07
Grade III	-/-	10/26 (38,5)	76/162 (46,9)	1,0 (ref.)	
	+/-	12/26 (46,2)	73/162 (45,1)	0,80 (0,33-1,97)	0,65
	+/+	4/26 (15,4)	13/162 (8,0)	0,43 (0,12-1,57)	0,24
	+/- + +/+	16/26 (61,5)	86/162 (53,1)	0,71 (0,30-1,65)	0,53
	**Alleles**				
	-	0,62	0,69	-	
	+	0,38	0,31	-	0,33
Grade IV	-/-	41/104 (39,4)	76/162 (46,9)	1,0 (ref.)	
	+/-	53/104 (50,9)	73/162 (45,1)	1,34 (0,80-2,26)	0,29
	+/+	10/104 (9,6)	13/162 (8,0)	1,42 (0,57-3,53)	0,48
	+/- + +/+	63/104 (60,6)	86/162 (53,1)	1,35 (0,82-2,24)	0,25
	**Alleles**				
	-	0,64	0,69	-	
	+	0,35	0,31	-	0,29

Table S2. Distribution of allele and genotype frequencies of polymorphism and statistical data obtained from the analysis of integrin *α2 BglII* for controls and astrocytomas patients (grade II, III or IV): Grade II astrocytoma and grade III

	**Number (%)**			
	**Astrocytoma**			
	**Grade II**	**Grade III**	**OR (95% CI)**	**P**
Integrin α2β1 BglII				
-/-	8/28 (28,6)	10/26 (38,5)	1,00 (ref.)	
+/-	14/28 (50,0)	12/26 (46,2)	1,45 (0,43-4,88)	0,76
+/+	6/28 (21,4)	4/26 (15,4)	1,87 (0,38-9,01)	0,69
+/- + +/+	20/28 (71,4)	16/26 (61,5)	1,56 (0,50-4,88)	0,56
Alleles				
-	0,53	0,62	-	
+	0,46	0,38	-	0,44
	**Grade II**	**Grade IV**		
-/-	8/28 (28,6)	41/104 (39,4)	1,0 (ref.)	
+/-	14/28 (50,0)	53/104 (50,9)	1,35 (0,51-3,53)	0,63
+/+	6/28 (21,4)	10/104 (9,6)	3,07 (0,86-10,89)	0,08
+/- + +/+	20/28 (71,4)	63/104 (60,6)	1,62 (0,65-4,04)	0,37
**Alleles**				
-	0,53	0,64	-	
+	0,46	0,35	-	0,12
	**Grade III**	**Grade IV**		
-/-	10/26 (38,5)	41/104 (39,4)	1,0 (ref.)	
+/-	12/26 (46,2)	53/104 (50,9)	0,92 (0,36-2,36)	1,00
+/+	4/26 (15,4)	10/104 (9,6)	1,64 (0,43-6,32)	0,47
+/- + +/+	16/26 (61,5)	63/104 (60,6)	1,04 (0,43-2,51)	1,00
**Alleles**				
-	0,62	0,64	-	
+	0,38	0,35	-	0,74

Table S3. Distribution of allele and genotype frequencies of polymorphism and statistical data obtained from the analysis of integrin $\alpha 2$ *BglII* by gender

	TOTAL NUMBER (%)			
**GENOTIPE**	**MALE**	**FEMALE**	**OR (95% CI)**	***P***
Integrin-/-				
Patients	38/97 (39,2)	21/61 (34,4)	1,0 (ref.)	
Controls	45/98 (46,4)	31/64 (48,4)	1,0 (ref.)	
Integrin+/-				
Patients	47/97 (48,5)	32/61 (52,5)	1,23 (0,61-2,47)	0,59
Controls	43/98 (43,9)	30/64 (46,9)	1,01 (0,53-1,95)	1,00
INTEGRINA+/+				
Patients	12/97 (12,4)	8/61 (13,1)	1,21 (0,43-3,42)	0,79
Controls	10/98 (10,2)	3/64 (4,7)	0,44 (0,11-1,71)	0,35

Table S4. Distribution of allele and genotype frequencies of polymorphism and statistical data obtained from the analysis of integrin $\alpha 2$ *BglII* by race

	TOTAL NUMBER (%)			
**GENOTIPE**	**WHITE**	**NOT WHITE**	**OR (95% CI)**	**P**
Integrin-/-				
Patients	49/120 (40,8)	10/38 (26,3)	1,00 (ref.)	
Controls	51/113 (42,5)	25/49 (51,0)	1,00 (ref.)	
Integrin+/-				
Pacients	56/120 (46,7)	23/38 (60,5)	2,01 (0,87-4,64)	0,11
Controls	55/113 (48,7)	18/49 (36,7)	0,67 (0,33-1,37)	0,28
Integrin+/+				
Patients	15/120 (12,5)	5/38 (13,1)	1,63 (0,48-5,53)	0,51
Controls	7/113 (6,2)	6/49 (12,2)	175 (0,53-5,75)	0,36

Table S5 – Association of *ITGA2* expression and clinicopathologic data in Low Grade Gliomas

		***ITGA2* expression – N (%)**		
		**High**	**Low**	**P Value**
Histology	Astrocytoma	97 (57.4%)	72 (42.6%)	0.001
	Oligoastrocytoma	66 (57.9%)	48 (42.1%)	
	Oligodendroglioma	69 (39.7%)	105 (60.3%)	
Grade	G2	98 (45.4%)	118 (54.6%)	0.029
	G3	134 (55.6%)	107 (44.4%)	
IDH status	Mutant	179 (42.8%)	239 (57.2%)	<0.001
	WT	78 (83.0%)	16 (17.0%)	
MGMT status	Methylated	197 (46.4%)	228 (53.6%)	<0.001
	Unmethylated	61 (67.8%)	29 (32.2%)	
TERT status	Mutant	75 (57.7%)	55 (42.3%)	0.794
	WT	91 (56.2%)	71 (43.8%)	
ATRX status	Mutant	95 (50.8%)	92 (49.2%)	0.835
	WT	162 (49.8%)	163 (50.2%)	
Original subtype	IDHumt-codel	52 (30.8%)	117 (69.2%)	<0.001
	IDHmut-non-codel	126 (50.8%)	122 (49.2%)	
	IDHwt	79 (83.2%)	16 (16.8%)	
Cecarelli	Classic-like	22 (95.7%)	1 (4.3%)	<0.001
	Codel	53 (30.6%)	120 (69.4%)	
	G-CIMP-high	120 (51.5%)	113 (48.5)	
	G-CIMP-low	6 (50.0%)	6 (50.0%)	
	Mesenchymal-like	42 (93.3%)	3 (6.7%)	
	PA-like	14 (53.8%)	12 (46.2)	

Table S6 – Association of *ITGA2* expression and clinicopathologic data in Glioblastomas

		ITGA2 expression – N (%)		
		**High**	**Low**	**P Value**
IDH status	Mutant	2 (18.2%)	9 (81.8%)	0.027
	WT	75 (52.8%)	67 (47.2%)	
MGMT status	Methylated	32 (60.4%)	21 (39.6%)	0.108
	Unmethylated	33 (45.8%)	39 (54.2%)	
TERT status	Mutant	17 (60.7%)	11 (39.3%)	0.018
	WT	0	5 (100.0%)	
ATRX status	Mutant	2 (22.2%)	7 (77.8%)	0.167
	WT	71 (51.1%)	68 (48.9%)	
Original subtype	Classical	28 (70.0%)	12 (30.0%)	0.001
	G-CIMP	1 (12.5%)	7 (87.5%)	
	Mesenchymal	30 (58.8%)	21 (41.2%)	
	Neural	13 (46.4%)	15 (53.6%)	
	Proneural	8 (26.7%)	22 (73.3%)	
Cecarelli	Classic-like	30 (63.8%)	17 (36.2%)	0.118
	G-CIMP-high	0	2 (100.0%)	
	G-CIMP-low	1 (20.0%)	4 (80.0%)	
	LGm6-GBM	6 (54.5%)	5 (45.5%)	
	Mesenchymal-like	25 (47.2%)	28 (52.8%)	

Table S7 – Cox regression analysis for *ITGA2* expression using overall survival as endpoint

			**95% confidence interval**		
		**Hazard ratio**	**Lower**	**Upper**	**P value**
Low grade glioma	Low expression	ref	ref	ref	
	High expression	2.361	1.633	3.412	<0.001
Glioblastoma	Low expression	ref	ref	ref	
	High expression	1.117	0.784	1.592	0.539
